# Olympic Elbow Comprising Lateral Epicondylitis, Cubital Tunnel Syndrome, and Distal Biceps Tendon Rupture

**DOI:** 10.7759/cureus.45902

**Published:** 2023-09-25

**Authors:** Pasquale Gencarelli, Rahul Mittal, Rosemary Yi, James M Lee

**Affiliations:** 1 Department of Orthopaedic Surgery, Rutgers Robert Wood Johnson Medical School, New Brunswick, USA; 2 Health Informatics, Rutgers University, Newark, USA; 3 Department of Orthopedic Surgery, Emanate Health Medical Group, West Covina, USA; 4 Department of Orthopaedic Surgery, Orange Orthopaedic Associates, West Orange, USA

**Keywords:** lateral epicondylitis elbow, operative fixation, olympic elbow, distal biceps rupture, cubital tunnel syndrome

## Abstract

There is a wide range of elbow injuries including chronic injuries such as lateral epicondylitis, medial epicondylitis, and cubital tunnel syndrome, or acute injuries such as elbow fracture-dislocations and distal biceps tendon ruptures. Combinations of acute and chronic elbow injuries have been reported including country club elbow and terrible triad of the elbow which are important to identify to properly treat. Thus, we report for the first time a new elbow injury triad termed Olympic elbow in a 65-year-old man comprising lateral epicondylitis, cubital tunnel syndrome, and a distal biceps tendon rupture. After initially failing conservative management, the patient was successfully treated with surgery and has returned to full physical activity at four and a half years postoperatively.

## Introduction

Elbow injuries encompass a wide range of conditions with some more common, such as lateral epicondylitis (tennis elbow), medial epicondylitis (golfer’s elbow), cubital tunnel syndrome, and traumatic elbow fracture-dislocations, and some less common, such as distal biceps tendon ruptures [1-5]. Combinations of these chronic and acute injuries have also been reported, including country club elbow consisting of medial and lateral epicondylitis, as well as the terrible triad of the elbow consisting of a radial head fracture, a coronoid process fracture, and a posterior elbow dislocation [6,7]. However, in this study, we propose a new condition termed Olympic elbow consisting of lateral epicondylitis, cubital tunnel syndrome, and a distal biceps tendon rupture. To date, no studies have clinically reported on this injury pattern or the surgical treatment of this condition. The objective of this study is to present the clinical outcomes of the case four and a half years after the surgical procedure.

## Case presentation

**Figure 1:**
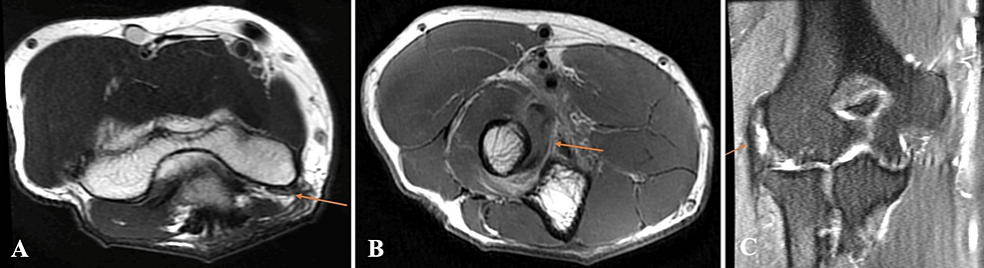
Preoperative magnetic resonance imaging without contrast of the right elbow. Coronal T1 view (A) with an arrow identifying inflammation of the ulnar nerve in the cubital tunnel. Axial T1 view (B) with an arrow identifying complete disruption of distal biceps tendon proximal to radial tuberosity. Coronal T2 view (C) with an arrow identifying severe partial tear of the common extensor tendon at the level of the lateral epicondyle.

A 65-year-old man with a past medical history of hypertension, gout, and hyperlipidemia presented to the clinic with a five-year history of chronic right elbow pain. The patient attributed the elbow pain to repetitive activities such as playing tennis and golf and working in construction when he was younger. The patient also reported a distinct event when he felt a pop in his right elbow while playing tennis with a resultant loss of elbow flexion strength a few months before surgical treatment. The patient had attempted prior physical therapy, two corticosteroid injections into the extensor carpi radialis brevis (ECRB) origin, rest, and non-steroidal anti-inflammatory drugs (NSAIDs) with only intermittent improvement of symptoms. Physical examination of the right upper extremity demonstrated tenderness to palpation along the lateral epicondyle and in the antecubital fossa at the site of distal biceps insertion. The patient had reproducible pain with resisted wrist extension over the lateral epicondyle and with resisted supination at the insertion of the distal biceps tendon. The elbow range of motion was 0-120 degrees of flexion, 70 degrees of supination, and 60 degrees of pronation. Strength was 4/5 for elbow flexion and 5/5 for elbow extension. A positive Tinel’s sign was elicited along the medial epicondyle with pain radiating to the ulnar fourth and fifth digits. Sensation from C5-T1 was intact. Radial and ulnar artery pulses were 3+. Magnetic resonance imaging (MRI) without contrast of the right elbow demonstrated complete disruption of the distal biceps tendon and severe partial tear of the common extensor tendon at the level of the lateral epicondyle (Figures 1A-1C). The signs and symptoms along with the physical examination and MRI findings confirmed the diagnosis of lateral epicondylitis, cubital tunnel syndrome, and distal biceps tendon rupture.

The patient was indicated for surgery as a result of his failure to improve with non-surgical measures. The procedure was performed using general anesthesia with the patient in the supine position. First, a 5 cm incision was made distal to the right elbow crease and medial to the mobile wad. The interval between pronator teres and brachioradialis was developed and dissection carried down to the distal biceps insertion on the radial tuberosity while protecting the lateral antebrachial cutaneous nerve. The remnant tendon was dissected off the radial tuberosity and prepared by taking 1 cm off the distal stump. The graft was prepared with a whipstitch using fiber wire. The radial tuberosity was denuded of the remnant soft tissue, drilled bicortically, and reamed 15 mm along the near cortex. EndoButton was attached to the suture construct of the whipstitch, delivered to the contralateral cortex, and flipped with the tendon reduced into the cortical window. Additional fixation was achieved with a 7 mm biocomposite interference screw. Attention was then turned to the ulnar nerve. An 8 cm incision was made posterior to the medial epicondyle spanning the triceps interval to the level of the flexor carpi ulnaris (FCU). The ulnar nerve was identified proximally in the triceps interval, dissected free, and fascial remnants around the nerve dissected down to the level of the FCU which was split proximally, freeing the nerve of adhesions. Flexion of the elbow demonstrated a non-subluxable ulnar nerve. Lastly, attention was turned to the lateral epicondyle. A 4 cm incision was made anterior to the lateral epicondyle and subcutaneous tissue was taken down to the junction of the extensor carpi radialis longus and extensor digitorum communis. This junction was incised revealing ECRB and denuded lateral epicondylitis tissue with the degenerative tissue subsequently removed. The incisions were then irrigated with normal saline, closed in layers, and sterilely dressed. The patient was placed in a well-padded posterior splint.

The patient did not receive any other postoperative treatments in the postoperative anesthesia care unit and was discharged home with our standard pain control protocol. The patient was not prescribed a course of postoperative antibiotics but did receive cefazolin 2 g intravenously immediately before the procedure. At the time of discharge, the patient’s sensory and motor functions were intact. The patient began physical therapy one week postoperatively and was prescribed protected weight-bearing lifting <5 lbs. At two weeks postoperatively the patient presented with mild swelling and tenderness along the lateral elbow incision with superficial serosanguinous drainage and scant pus from the biceps incision concerning for a superficial wound infection. The patient was prescribed cephalexin 500 mg PO twice a day for one week with complete resolution of symptoms at three weeks postoperatively. At eight weeks postoperatively the patient had an elbow range of motion of 0-145 degrees of flexion, 80 degrees of supination, and 75 degrees of pronation with further progression of strengthening modalities. At six months postoperatively the patient returned to full physical activity as tolerated. At the most recent follow-up of four and a half years, the patient had a full right upper extremity range of motion, motor strength of 5/5, and intact sensation.

## Discussion

To date, no studies have been reported on the occurrence and treatment of concurrent lateral epicondylitis, cubital tunnel syndrome, and distal biceps tendon rupture. Given the severity of the three injuries, Olympic elbow seemed a fitting title as the pathology encompasses the elbow globally.

The etiology of the three individual injuries has been previously reported and is well described [8-10]. However, in the case of Olympic elbow, we propose the etiology of the injuries is sequential secondary to tendon degeneration with chronic inflammation in contrast to the acute pathology observed in the terrible triad of the elbow. It has been reported by Abate et al. that microrupture of tendon fibers results in the release of several inflammatory cytokines and subsequent tendon degeneration [11]. Neural ingrowth that accompanies the neovessels results in pain and neurogenic-mediated inflammation [11]. In our patient, initially, the patient’s lateral epicondylitis affecting the ECRB was likely caused by repetitive microtrauma from excessive gripping or wrist extension, radial deviation, and/or forearm supination secondary to playing tennis [10]. Second, as a result of years of chronic inflammation in addition to repetitive forehand tennis strikes and playing golf stressing the medial elbow, cubital tunnel syndrome likely developed from compression of the ulnar nerve under Osborn’s ligament [8]. Lastly, the combination of chronic inflammation from the aforementioned two injuries in addition to repetitive pronosupination from tennis and golf likely resulted in mechanical attrition of the distal biceps tendon. Ultimately, eccentric contraction of the biceps with the extension of the arm during a forehand strike while playing tennis resulted in acute overload and failure of the chronically degenerated distal biceps tendon causing it to rupture.

Currently, first-line treatment for lateral epicondylitis is non-operative including rest, NSAIDs, and activity modification. If non-operative therapy fails, usually between six and 12 months, and symptoms persist, surgical treatment is pursued. A study by Knutsen et al. identified factors associated with failure of non-operative treatment of lateral epicondylitis and identified a history of prior corticosteroid injection as well as duration of symptoms >12 months as independent predictors of non-operative treatment failure [12,13]. Moreover, although rare, reports of tendon rupture after corticosteroid injection have been reported in the literature [13,14]. Our patient received two corticosteroid injections into the ECRB origin, and his lateral epicondylitis symptoms began approximately five years before his ultimate surgical intervention with interim development of cubital tunnel syndrome and distal biceps tendon rupture. Thus, early identification of physically active elderly patients with chronic symptoms and risk factors for non-operative treatment failure of lateral epicondylitis may benefit from more aggressive early interventions to prevent the progression of the condition, or worse, the development of Olympic elbow.

However, if Olympic elbow occurs, surgical intervention is a viable treatment. Our patient underwent the above-mentioned procedures and is overall pleased with his recovery. At the most recent follow-up of four and a half years, the patient reported a Quick Disabilities of the Arm, Shoulder & Hand score of 15.9 and has returned to playing pickleball and golf weekly as well as weightlifting three times a week with no residual pain in his right elbow.

## Conclusions

With the importance of properly diagnosing patients with combination elbow injuries to provide optimal care, physicians must be made aware of new injury patterns. Our case highlights the identification of a new chronic elbow injury triad termed Olympic elbow. Surgeons should be mindful of this condition when counseling physically active elderly patients on non-operative treatment of lateral epicondylitis. In patients who develop Olympic elbow, surgical treatment provides acceptable functional outcomes. Future research should focus on the epidemiology and associated risk factors of Olympic elbow to better guide prevention strategies.
